# Quinin and Urea as Local Anesthetics

**Published:** 1910-06-15

**Authors:** 


### QUININ AND UREA AS LOCAL ANESTHETICS.

Quinin and urea hydrochlorid is a double salt of quinin and urea made by dissolving quinin hydrochlorid in hydrochloric acid, adding pure urea, filtering the mixture through glass wool and allowing it to crystalize. It is soluble in its own weight of water and in alcohol. It has the actions of quinin, is non-irritating when injected hypodermically, and produces local anesthesia, lasting in some instances several days, depending on the strength of the solution.

Dr. Hoy asserts that he has used quinin for fifteen years by external application on denuded surfaces (not hypodermically), and believes that Dr. Sylvester of Ohio, from whom he obtained the suggestion, is entitled to credit for priority.

Helmholtz used it unsuccessfully in watery solution as a spray in hay fever. Fulton (*The Journal*, July 30, 1904, p. 1393) attributed this failure to the fact that watery solutions are transient in their effect in relieving the coryza and the other symptoms, and he therefore followed the spray with an ointment of quinin in white petrolatum, 30 grains to the ounce. This, he asserted, gave continued relieve from the symptoms while it was employed. Thibault (*Jour. Ark. Med. Soc.*, September, 1907; abstr. in *The Journal* Nov. 16, 1907, p. 1719) also reports its efficiency for anesthesia when used locally on cotton or gauze on mucous membranes or denuded surfaces in strength of 15 or 20 per cent. In a case of strangulated hernia in an aged woman, reported later (*The Journal*, April 23, 1910, p. 1375), he used the infiltration anesthesia in 0.25 per cent. solution. On manipulating the viscera and peritoneum pain was caused. Some of the same solution was poured on these structures and the operation completed without pain. He suggests that it might be used in more extensive abdominal operations, involving manipulation of the viscera and peritoneum.

Quinin has been used hypodermically for a number of years in the treatment of malaria, but its hypodermic use as a local anesthetic is more recent. In the communication mentioned above, H. Thibault reported on its hypodermic use both experimentally on himself and practically in the amputation of a finger, in the removal of a large fatty tumor and in the divulsion of the sphincter ani for the removal of a foreign body. He first used quinin and urea hydrochlorid, but stated that any of the salts of quinin could be used for this purpose, in 1 to 2 per cent. solution, which might be boiled any number of times without destroying its efficiency. He said: "It is safe, and the anesthesia lasts from one to six hours." In August, 1908, E. J. Brown (*The Journal*, Aug. 8, 1908, p. 496) reported the use of 3 per cent. solution by hypodermic injection in the removal of the tonsils, giving perfect anesthesia for a prolonged period after the operation and limiting the bleeding during the operation. Dr. V. M. Griswold, of Fredonia, N. Y., then called attention (*The Journal*, Aug. 22, 1908) to the fact that he reported on the hypodermic use of quinin as an efficient local anesthetic and much safer than cocain, in July, 1896, before the Chautauqua County (New York)

Medical Society (*Buffalo Med. Jour.* August, 1896, p. 32). Dr. Griswold claims that his use of quinin as a local anesthetic was the result of experiments with various substances in the endeavor to find one equally efficient, but less dangerous than cocain.

Dr. John A. Wyeth (*New York Policlinic Journal,* January, 1908) reports on the use of quinin and urea for this purpose in an article entitled "A New Local Anesthetic of Great Value." On account of two cases of severe sloughing after deep infiltration Wyeth has abandoned its use, and is now trying a different formula which he will report on later.

The first attempt to explain the anesthetic effect of the solution is found in a paper by E. F. McCampbell (*The Journal,* March 16, 1907, p. 919), who found that quinin hydrochlorid had the power of causing temporary paralysis in experimental animals, which he believed to be due to its doagulating action on the protoplasm of the peripheral nerves. He found that atrophy of the extremities resulted after large doses.

Hertzler, Brewster and Rogers (*The Journal,* Oct. 23, 1909, p. 1393) confirmed Thibault's statements, but found that in the hernia operations healing of the external wound was somewhat delayed on account of the thickening of its edges, which proved to be due to an excessive fibrinous exudate produced by the 1 per cent. solution. When 0.25 per cent. instead of 1 per cent. solutions were used the fibrinous exudate was reduced and healing was not perceptibly delayed. Solutions of the 0.25 per cent. strength produced perfect though less prompt and lasting anesthesia. Hertzler, Brewster and Rogers agreed with Thibault that a 10 or 20 per cent. solution should be used externally or on mucous membranes, and with Brown that in tonsillectomy a 3 per cent. solution is best. They recommend this form of anesthesia especially in operation about the anus, as in hemorrhoids and fistulas, in which prolonged anesthesia from seven to ten days is particularly desirable.

They have found that it is preferable to dissolve the quinin and urea hydrochlorid in sterile water instead of salt solution; the former gives a more lasting anesthesia, and the latter has no advantage. They recommend it as being harmless in almost any amount. There are, however, many reports of untoward effects from the hypodermic use of quinin in malaria and other affections in which it has been used. But in the weak solutions of 0.25 to 1 or even 3 per cent. recommended, unpleasant effects are unlikely to occur, unless there is an idiosyncrasy to quinin in any amount. Quinin and urea hydrochlorid, therefore, seems to be a valuable addition to the list of local anesthetics, on account of its cheapness, its simplicity of preparation and use, its safety, its hemostatic effect and the prolonged anesthesia produced.—*A. M. A. Journal.*

